# Prognostic Value of the SUVmax–IPI Composite Score on Overall Survival in Metastatic Prostate Cancer

**DOI:** 10.3390/jcm15072655

**Published:** 2026-03-31

**Authors:** Emine Türkmen, Atike Pınar Erdoğan, Mustafa Şahbazlar, Gözde Mütevelizade, Ferhat Ekinci

**Affiliations:** 1 Department of Medical Oncology, Faculty of Medicine, Manisa Celal Bayar University, 45047 Manisa, Türkiye; 2 Department of Nuclear Medicine, Faculty of Medicine, Manisa Celal Bayar University, 45047 Manisa, Türkiye

**Keywords:** metastatic prostate cancer, PSMA PET/CT, SUVmax, inflammatory prognostic index, overall survival, prognostic biomarker

## Abstract

**Objective:** This study aimed to evaluate the prognostic value of the SUVmax–IPI composite score, generated by integrating the maximum standardized uptake value (SUVmax) derived from metastatic ^68^Ga-PSMA PET/CT imaging with the inflammatory prognostic index (IPI), in predicting overall survival in patients with metastatic prostate cancer. **Materials and Methods:** This retrospective, single-center cohort study included 146 patients diagnosed with metastatic prostate adenocarcinoma between 2009 and 2025. Among them, 125 patients with available PET/CT imaging were included in the SUVmax–IPI analysis. The composite score was calculated by multiplying the metastatic SUVmax value by the IPI. The optimal cut-off value was determined using receiver operating characteristic curve analysis. Overall survival was evaluated using the Kaplan–Meier method and compared using the log-rank test. Independent prognostic factors were identified using multivariable Cox proportional hazards regression analysis with a forward (stepwise) selection approach. **Results:** Using the predefined cut-off value (82), the median overall survival was 125 months in patients with SUVmax–IPI ≤ 82 and 19 months in those with SUVmax–IPI > 82 (log-rank *p* = 0.001). In the forward multivariable Cox regression model, SUVmax–IPI > 82 remained independently associated with worse overall survival after adjustment for ALP, AST, PSA nadir, and androgen deprivation modality (hazard ratio [HR]: 7.92; 95% confidence interval [CI]: 2.97–21.10; *p* < 0.001). **Conclusions:** The SUVmax–IPI composite score, integrating PSMA PET/CT-derived metabolic tumor activity with systemic inflammatory burden, is independently associated with overall survival in metastatic prostate cancer. These findings suggest that combining metabolic and inflammatory parameters may enhance prognostic stratification beyond conventional clinical and biochemical markers.

## 1. Introduction

Prostate cancer is among the most frequently diagnosed malignancies in men, and mortality increases significantly in advanced stages [1,2]. Although some patients exhibit prolonged responses to androgen deprivation therapy (ADT), others develop early treatment resistance, leading to rapid disease progression and increased mortality [3,4]. This marked heterogeneity has heightened the need for more accurate and biologically grounded risk stratification in metastatic patients.

In clinical practice, conventional prognostic indicators such as prostate-specific antigen (PSA) levels, Gleason score, metastatic disease pattern, hemoglobin, lactate dehydrogenase (LDH), and alkaline phosphatase (ALP) are widely used [5,6,7]. However, these parameters do not always adequately reflect the complex nature of tumor biology. Although the lowest PSA level achieved during treatment (PSA nadir) has been shown to be associated with overall survival [8], its predictive value for identifying aggressive biology in the pretreatment setting remains limited. Therefore, there is an ongoing need for novel biomarkers that can more comprehensively reflect prognosis in mPCa.

Prostate-specific membrane antigen (PSMA) targeted PET/CT imaging has gained an important role in the detection and staging of metastatic disease by providing higher sensitivity compared to conventional imaging modalities [9,10]. The overexpression of PSMA in prostate cancer cells enables PET-based measurements to more directly reflect tumor biology [11]. In this context, the maximum standardized uptake value (SUVmax) represents the highest metabolic activity within the tumor and has emerged as a practical and easily applicable parameter in clinical settings [12]. However, inconsistent findings have been reported regarding the prognostic value of PSMA PET/CT-derived metabolic and volumetric parameters, and it has been suggested that these indicators alone may be insufficient to fully explain the heterogeneity of tumor biology [13,14].

Although SUVmax is the most readily available parameter from PSMA PET/CT, volumetric parameters such as PSMA tumor volume (PSMA-TV) and total lesion PSMA (TL-PSMA) have also been investigated as prognostic markers in metastatic prostate cancer. However, the independent prognostic value of these parameters remains controversial. For instance, Seifert et al. demonstrated that PSMA PET-derived organ-specific tumor volumes were associated with survival, but their prognostic significance diminished after adjustment for clinical variables [15]. Moreover, PSMA expression can be heterogeneous across metastatic lesions and may change dynamically under androgen receptor-targeted therapies, potentially limiting the prognostic accuracy of a single-time-point measurement.

The central role of systemic inflammation in cancer progression has become increasingly recognized. The inflammatory response plays a critical role in tumor proliferation, invasion, metastasis, angiogenesis, and treatment resistance [16,17]. Accordingly, various inflammation-based prognostic indices derived from C-reactive protein (CRP), albumin, and peripheral blood cell distributions have been developed. The Inflammatory Prognostic Index (IPI), which integrates CRP, neutrophil-to-lymphocyte ratio, and albumin parameters, has demonstrated clinical value in predicting survival across different solid tumors [18,19,20]. In addition, other inflammation-based indices such as the Immune-Based Prognostic Index (IBI) and the Systemic Inflammation Response Index (SIRI) have also been investigated for prognostic assessment [17,20].

In this context, SUVmax–IPI is a composite biomarker based on the integration of SUVmax derived from PSMA PET/CT with IPI reflecting systemic inflammation. Although the prognostic value of this approach has been reported in different solid tumor types [18], no study has evaluated the prognostic performance of SUVmax–IPI in metastatic prostate cancer.

In the present study, we evaluated the prognostic value and clinical applicability of the SUVmax–IPI composite score in predicting overall survival in patients with metastatic prostate cancer.

## 2. Materials and Methods

This retrospective, single-center cohort study was based on the analysis of patients diagnosed with metastatic prostate adenocarcinoma and followed at the Medical Oncology Clinic of Manisa Celal Bayar University Faculty of Medicine between 2009 and 2025. The study protocol was approved by the institutional ethics committee. The requirement for written informed consent was waived by the ethics committee due to the retrospective design of the study.

Patients aged 18 years or older with histopathologically confirmed prostate adenocarcinoma who were diagnosed at the metastatic stage or developed metastatic disease during follow-up and received systemic treatment for metastatic disease (androgen deprivation therapy [ADT] ± chemotherapy or next-generation hormonal agents) were included. Patients with incomplete clinical, laboratory, or imaging data, as well as those without evidence of metastatic disease, were excluded. The final analysis was performed on a total of 146 patients.

For each patient, demographic characteristics, comorbidities, ECOG performance status, smoking history, family history of malignancy, Gleason score, TNM stage, metastatic sites (bone, liver, lung, and non-regional lymph nodes), CHAARTED tumor volume classification, local treatments (radical prostatectomy, radiotherapy, and transurethral resection of the prostate [TURP]), and first-line systemic treatment regimens were recorded.

Laboratory assessments included complete blood count and routine biochemical parameters, as well as clinically relevant inflammatory and prognostic biomarkers such as C-reactive protein (CRP), albumin, alkaline phosphatase (ALP), aspartate aminotransferase (AST), lactate dehydrogenase (LDH), and PSA. PSA at diagnosis, PSA at the time metastatic disease was detected, and the lowest PSA level achieved during treatment (PSA nadir) were also evaluated.

All patients underwent ^68^Ga-PSMA-11 PET/CT imaging at diagnosis or prior to systemic treatment. Imaging was performed approximately 60 min after intravenous administration of the radiopharmaceutical, using low-dose CT for attenuation correction and anatomical localization. Images were evaluated according to standard clinical protocols by experienced nuclear medicine specialists. Metabolic parameters derived from PSMA PET/CT included maximum standardized uptake value (SUVmax), PSMA tumor volume (PSMA-TV), and total lesion PSMA activity (TL-PSMA). Measurements related to the primary tumor were obtained from the prostate lesion. In patients with metastatic disease, quantitative analyses were based on the SUVmax value of the metastatic lesion with the highest uptake. All imaging procedures were performed at the same center using the same scanner and reconstruction algorithms. In routine clinical practice, detailed lesion-based quantitative parameters such as SUVmax values are not systematically included in standard reports. Therefore, all PET/CT datasets were retrospectively re-evaluated. SUVmax measurements were manually obtained from the original PET/CT images. For each patient, the lesion demonstrating the highest SUVmax value was identified and recorded for analysis in order to reflect the maximum metabolic tumor activity.

The SUVmax–IPI composite score analysis was conducted in 125 patients with available PSMA PET/CT imaging. Patients without PET/CT imaging were excluded from the SUVmax–IPI analysis but were included in the overall clinical and survival analyses of the full cohort.

Inflammation-based indices reflecting systemic inflammatory status were calculated using laboratory data as follows: neutrophil-to-lymphocyte ratio (NLR) = neutrophils/lymphocytes; inflammatory prognostic index (IPI) = (CRP × NLR)/albumin; systemic inflammation response index (SIRI) = (neutrophils × monocytes)/lymphocytes; immune-based prognostic index (IBI) = (CRP × neutrophils)/lymphocytes; and the albumin-to-alkaline phosphatase ratio (ALB/ALP) was also calculated. The primary biomarker of interest, the SUVmax–IPI composite score, was calculated by multiplying the metastatic SUVmax value by the IPI (SUVmax–IPI = metastatic SUVmax × IPI).

The primary endpoint of the study was overall survival. Overall survival was defined as the time from the date of diagnosis of metastatic disease to death from any cause or the date of last follow-up. Continuous variables were presented as median and interquartile range (IQR), and categorical variables were expressed as numbers and percentages. Between-group comparisons were performed using the Mann–Whitney U test, chi-square test, or Fisher’s exact test, as appropriate. The discriminative ability of biomarkers for mortality was evaluated using receiver operating characteristic (ROC) curve analysis with the Youden index to determine optimal cut-off values. Survival analyses were performed using the Kaplan–Meier method and compared using the log-rank test. Univariable Cox regression analyses were first conducted to identify prognostic determinants of mortality; variables with *p* < 0.10 in univariable analysis or considered clinically relevant were included in the multivariable Cox regression model. To avoid overfitting, given the limited number of events relative to the number of candidate variables, a forward stepwise selection approach was used for the final multivariable model, with entry and retention thresholds set at *p* < 0.10. A two-sided *p* value < 0.05 was considered statistically significant. The proportional hazards assumption was tested using Schoenfeld residuals, and no significant violation was observed.

## 3. Results

### 3.1. Patient Characteristics

A total of 146 patients with histopathologically confirmed prostate adenocarcinoma who were diagnosed at the metastatic stage or developed metastatic disease during follow-up were included in the study. At the end of the median follow-up period, 113 patients (77.4%) were alive, while 33 (22.6%) were recorded as deceased. The median age at diagnosis was 68 years (IQR: 62–72.3).

Regarding comorbidities, 27.4% of the patients had coronary artery disease, 48.6% had hypertension, and 22.8% had diabetes mellitus. Statin use was reported in 22.6% of patients, and 27.4% were receiving aspirin therapy. In terms of smoking history, 20.7% had never smoked, 44.1% were former smokers, and 35.2% were current smokers. Among smokers, the median smoking exposure was 30 pack-years (IQR: 8–50).

Histopathological evaluation showed the following Gleason score distribution: 3 + 4 in 7.5% of patients, 4 + 3 in 17.5%, 4 + 4/3 + 5/5 + 3 in 14.0%, and 4 + 5/5 + 4/5 + 5 in 60.1%. Gleason score data were unavailable in four patients (2.7%).

At initial clinical staging, 2.7% of patients were classified as T1, 12.5% as T2, 18.6% as T3, and 61.4% as T4. Regional lymph node positivity at diagnosis was detected in 69% of patients.

When patterns of distant metastatic involvement were evaluated, bone was the most common site of metastasis, observed in 76.7% of patients. Non-regional lymph node metastasis was present in 82.9% of patients. Lung and liver metastases were observed in 8.9% and 6.8% of patients, respectively. Among patients with bone metastases, 17.8% had 1–4 bone metastatic lesions, while 58.9% had ≥4 bone metastases and/or involvement beyond the axial skeleton. According to the CHAARTED tumor volume classification, 64.4% of patients were categorized as having high-volume disease and 35.6% as having low-volume metastatic disease.

Regarding local treatment directed at the primary tumor, 35.7% of patients had undergone radical prostatectomy, 12.6% had received radiotherapy, and 1.8% had undergone transurethral resection of the prostate (TURP). Following radical prostatectomy, 18.7% of patients received adjuvant radiotherapy and 7.5% received salvage radiotherapy. The proportion of patients receiving adjuvant androgen deprivation therapy (ADT) after local treatment was 31.1%.

First-line systemic treatments for metastatic disease consisted of LHRH analog monotherapy (15.1%) or LHRH analog combined with docetaxel (31.5%), abiraterone (24.0%), enzalutamide (22.6%), apalutamide (2.1%), or bicalutamide (4.1%). Responses to first-line therapy were distributed as follows: complete response in 16.4%, partial response in 35.6%, stable disease in 44.5%, and progressive disease in 3.4% of patients.

At the initiation of first-line systemic therapy for metastatic disease, ECOG performance status was classified as ECOG 0 in 15.1% of patients, ECOG 1 in 66.4%, ECOG 2 in 16.4%, and ECOG 3 in 2.1%.

The demographic, clinical, and treatment-related characteristics of the 125 patients with available PSMA PET/CT data are summarized in Table 1 according to SUVmax–IPI subgroups.

### 3.2. Survival Outcomes

**Figure 1:**
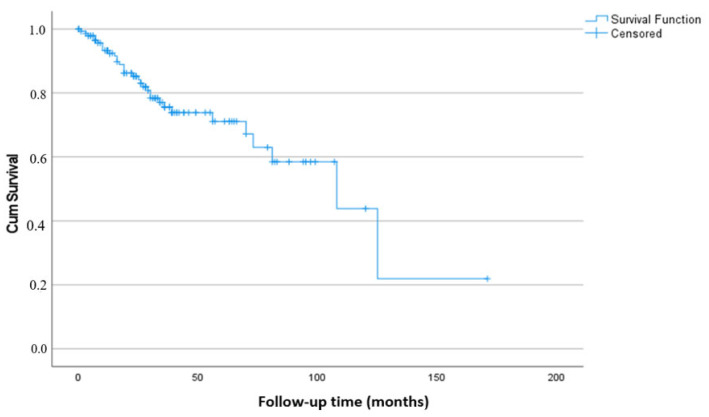
Kaplan–Meier analysis of overall survival in patients with metastatic prostate cancer (n = 146). The median overall survival was 108 months (95% CI: 57.4–158.6).

Overall survival (OS) was defined as the time from the date of metastatic disease diagnosis to death from any cause or the date of last follow-up. In patients without metastasis at initial diagnosis who subsequently developed metastatic disease during follow-up, OS was calculated from the date of first radiologically confirmed metastasis. Survival curves were generated using the Kaplan–Meier method. According to Kaplan–Meier analysis, the median overall survival for the entire cohort was 108 months (SE: 25.8; 95% CI: 57.4–158.6). The estimated 1-, 3-, 5-, and 10-year overall survival rates were 93.3% (SE: 2.2), 75.6% (SE: 4.3), 71.1% (SE: 5.2), and 43.9% (SE: 14.0), respectively (Figure 1).

### 3.3. Cox Regression Analyses

Clinical, biochemical, inflammatory, and imaging-based variables associated with overall survival were evaluated using univariable and multivariable Cox proportional hazards regression analyses.

In univariable analyses, alkaline phosphatase (ALP), lactate dehydrogenase (LDH), serum albumin, hemoglobin, and nadir prostate-specific antigen (PSA nadir) levels were significantly associated with mortality risk. Among clinical variables, ECOG performance status and the presence of liver metastasis emerged as factors adversely affecting overall survival. Additionally, SUVmax-IPI as a continuous variable showed a significant association with mortality (HR: 1.003; 95% CI: 1.002–1.005; *p* < 0.001) (Table 2). The complete results of the univariable and multivariable Cox regression analyses are presented in Supplementary Table S1.

Variables that were significant in univariable analyses and/or considered clinically relevant were included in multivariable Cox regression analyses. Multivariable analyses were performed using the results of the forward (stepwise) model, taking into account the statistical robustness of the model.

In the model constructed using the enter method, ALP, AST, albumin, LDH, hemoglobin, first-line treatment categories for metastatic disease, LHRH/orchiectomy status, nadir PSA, metastatic SUVmax > 10.7, ALB/ALP ≤ 0.04, IBI > 12.1, De Ritis ratio > 1.3, IPI > 3, and metastatic TL-PSMA > 13.9 were simultaneously included. In this model, only nadir PSA remained significantly associated with mortality (*p* < 0.001).

Considering the number of events and model stability, the final forward (stepwise) model identified ALP, AST, nadir PSA, LHRH/orchiectomy status, and SUVmax–IPI > 82 as independent predictors of overall survival (Table 3).

In this model, ALP (*p* = 0.032; HR = 1.002), AST (*p* = 0.035; HR = 1.031), and nadir PSA (*p* < 0.001; HR = 1.006) were identified as biochemical markers significantly associated with increased mortality risk.

LHRH/orchiectomy status retained its independent prognostic significance in the forward model. Patients who underwent orchiectomy had a significantly higher risk of mortality compared with those receiving goserelin treatment (*p* = 0.004; HR = 10.177; 95% CI: 2.064–50.174); however, the wide confidence interval should be interpreted cautiously given the small number of patients in this subgroup.

SUVmax–IPI > 82 was identified as a strong and independent prognostic factor in the forward model, increasing mortality risk by approximately eightfold (*p* < 0.001; HR = 7.917; 95% CI: 2.971–21.098) (Table 3).

### 3.4. Development and Prognostic Value of the SUVmax–IPI Composite Score

The SUVmax–IPI composite score was calculated in 125 patients with available PSMA PET/CT data. SUVmax–IPI was derived by multiplying the highest SUVmax value among metastatic lesions detected on ^68^Ga-PSMA PET/CT by the concurrently calculated inflammatory prognostic index (IPI) (SUVmax–IPI = metastatic SUVmax × IPI).

The discriminatory ability of SUVmax–IPI for predicting overall survival was evaluated using receiver operating characteristic (ROC) curve analysis. The area under the curve (AUC) was 0.549 (95% CI: 0.458–0.638). Although the overall discriminatory performance was limited, the optimal cut-off value determined using the Youden index was 82. Based on this threshold, patients were categorized into two groups: SUVmax–IPI ≤ 82 (n = 113) and SUVmax–IPI > 82 (n = 12).

#### 3.4.1. Clinical, Laboratory, and PSMA PET/CT Findings According to SUVmax–IPI Subgroups

No statistically significant differences were observed between the SUVmax–IPI subgroups in terms of age at diagnosis, comorbidities (coronary artery disease, hypertension, diabetes mellitus), statin and aspirin use, smoking history, Gleason score distribution, clinical T/N/M stage at diagnosis, metastatic involvement patterns, CHAARTED tumor volume classification, history of local treatment to the primary tumor, or first-line systemic therapy regimens for metastatic disease (all *p* > 0.05) (Table 1). Response rates to first-line therapy were also comparable between the groups.

In contrast, significant differences were observed in biochemical and imaging parameters (Table 4). Patients with SUVmax–IPI > 82 had significantly higher levels of alkaline phosphatase (ALP), C-reactive protein (CRP), and lactate dehydrogenase (LDH), whereas serum albumin and hemoglobin levels were significantly lower (all *p* < 0.05). Absolute neutrophil counts were also significantly elevated in the high SUVmax–IPI group.

Regarding PSA parameters, PSA levels at the time of metastatic diagnosis and nadir PSA values were significantly higher in patients with SUVmax–IPI > 82 (*p* < 0.05). While primary tumor SUVmax, PSMA-TV, and TL-PSMA values were similar between groups, metastatic lesion SUVmax and metastatic TL-PSMA values were significantly higher in the high SUVmax–IPI group (Table 4). The complete comparison of laboratory and PSMA PET/CT parameters is presented in Supplementary Table S2.

#### 3.4.2. Kaplan–Meier Overall Survival Analysis According to SUVmax–IPI

**Figure 2:**
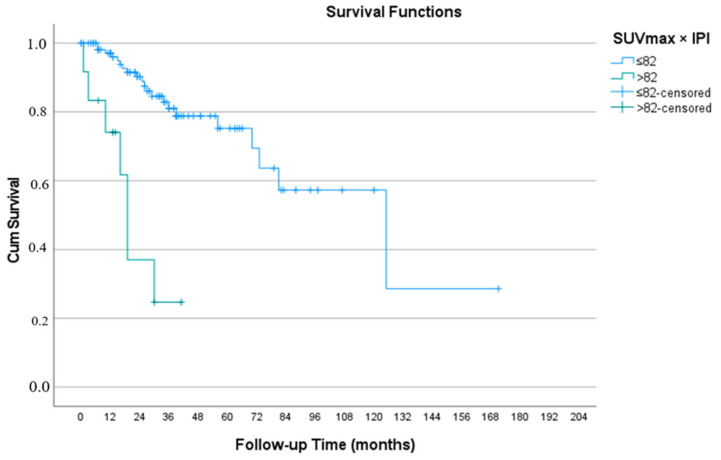
Kaplan–Meier overall survival curves according to SUVmax–IPI ≤ 82 and >82 subgroups. Patients with SUVmax–IPI ≤ 82 had significantly longer overall survival than those with SUVmax–IPI > 82 (log-rank *p* = 0.001). Median overall survival was 125 months and 19 months, respectively.

According to the Kaplan–Meier analysis based on the predefined SUVmax–IPI cut-off value, a statistically significant difference in overall survival was observed between the groups (log-rank *p* = 0.001; Figure 2).

The median overall survival was 125 months in patients with SUVmax–IPI ≤ 82, whereas it was 19 months in those with SUVmax–IPI > 82.

In the SUVmax–IPI ≤ 82 group, the 1-, 3-, 5-, and 10-year overall survival rates were 97.1%, 81.1%, 75.2%, and 57.3%, respectively. In contrast, in the SUVmax–IPI > 82 group, the 1- and 3-year overall survival rates were 74.1% and 24.7%, respectively.

Although the area under the curve (AUC) obtained from ROC analysis was modest, survival-based analyses demonstrated a clear separation between the groups. This finding suggests that SUVmax–IPI may serve as a clinically meaningful time-dependent prognostic biomarker in metastatic prostate cancer.

## 4. Discussion

In this study, we comparatively evaluated the prognostic roles of metabolic and volumetric imaging parameters derived from ^68^Ga-PSMA PET/CT and laboratory-based biomarkers reflecting systemic inflammation in patients with metastatic prostate cancer. Our findings demonstrated that metabolic (SUVmax) and volumetric PSMA PET/CT parameters alone were not independently associated with overall survival. In contrast, the composite SUVmax–IPI score, generated by integrating SUVmax with the inflammatory prognostic index (IPI), retained independent prognostic significance in both the conventional multivariable model and the forward (stepwise) analysis (HR: 7.92; 95% CI: 2.97–21.10; *p* < 0.001). These results suggest that prognosis in metastatic prostate cancer cannot be adequately explained by single parameters and that integrated models combining tumor biology and host inflammatory response provide more robust prognostic information.

The prognostic value of PSMA PET/CT-based volumetric parameters remains controversial in the literature. Seifert et al. demonstrated that PSMA PET/CT-derived organ-specific tumor volumes were associated with survival in metastatic prostate cancer; however, their independent prognostic value diminished when incorporated into multivariable models together with clinical variables [15]. This suggests that the prognostic performance of PSMA-based measurements may be highly dependent on tumor burden and clinical context.

Moreover, PSMA expression in metastatic disease may exhibit substantial inter-lesional heterogeneity, potentially limiting the accuracy of volume-based prognostic approaches [21]. Rosar et al. reported that enzalutamide treatment may induce upregulation of tumoral PSMA expression and that PSMA signal intensity can change dynamically in response to therapy [21]. These findings imply that a single-time-point SUVmax measurement may not fully capture the dynamic biological behavior of metastatic prostate cancer.

Consistent with these observations, volumetric PSMA parameters and SUVmax alone did not demonstrate independent prognostic significance in our cohort. SUVmax reflects the highest metabolic activity within a lesion; however, it does not account for total tumor burden, lesion heterogeneity, or microscopic disease. Therefore, integrating metabolic imaging parameters with inflammatory biomarkers may provide a more comprehensive representation of tumor biology.

The role of systemic inflammation in prostate cancer progression is increasingly recognized. Abbas et al. highlighted the central role of the inflammatory microenvironment in metastasis and treatment resistance [17], while Templeton et al. demonstrated in a large meta-analysis that the neutrophil-to-lymphocyte ratio is an independent prognostic factor across solid tumors [16].

Nevertheless, inflammation-based scores alone may not fully reflect the entire prognostic spectrum. Fan et al. reported that the systemic immune-inflammation index (SII) had limited prognostic value when used alone in metastatic castration-resistant prostate cancer but improved prognostic discrimination when integrated with clinical parameters [22]. Similarly, Mitsui et al. showed that combining the CRP/albumin ratio with time to castration resistance enhanced prognostic prediction compared to inflammatory markers alone [23]. These findings support the concept that integrated models encompassing both tumor-related and host-related factors may offer superior prognostic performance.

The integration of metabolic imaging parameters and inflammatory biomarkers has been successfully applied in other solid tumors. The prognostic value of the SUVmax–IPI composite score has been demonstrated in metastatic non-small-cell lung cancer patients receiving nivolumab [18]. The prognostic significance of IPI has also been reported in small-cell lung cancer [19]. In the original study defining IPI, its prognostic utility in non-small-cell lung cancer was emphasized [20].

Our study is among the first to demonstrate that this integrated approach is also applicable in metastatic prostate cancer and that the SUVmax–IPI composite score can serve as a strong prognostic biomarker. In the forward multivariable analysis, SUVmax–IPI > 82, ALP, AST, PSA nadir, and the modality of androgen deprivation therapy were identified as independent prognostic factors. Although LDH was significant in the univariable analysis, it did not retain independent significance in the final multivariable model. LDH is known to reflect cellular proliferation and overall tumor burden, while ALP particularly represents osteoblastic activity and metastatic spread in bone-dominant disease; both parameters have previously been incorporated into prognostic models in metastatic prostate cancer [22]. The inclusion of PSA nadir in the model is consistent with the literature, as it serves as a dynamic indicator of treatment response [6,7]. Notably, despite the presence of these established biochemical and clinical markers, SUVmax–IPI maintained a strong independent prognostic effect (HR: 7.92; *p* < 0.001), suggesting that the integration of metabolic tumor activity with systemic inflammatory status provides additional and clinically meaningful prognostic value.

The prognostic superiority of SUVmax–IPI was clearly demonstrated in Kaplan–Meier analyses (Figure 2). Patients with SUVmax–IPI ≤ 82 had a median overall survival of 125 months, compared with 19 months in those with SUVmax–IPI > 82 (log-rank *p* = 0.001). Although the AUC derived from ROC analysis was modest, the pronounced separation of survival curves indicates that SUVmax–IPI provides clinically meaningful time-dependent prognostic information. The integration of metabolic tumor activity and systemic inflammatory burden may therefore enable more biologically coherent risk stratification in metastatic prostate cancer. Early identification of high-risk patients may facilitate individualized treatment intensification and improved clinical decision-making.

The association between orchiectomy and increased mortality risk in the forward model warrants cautious interpretation. Orchiectomy is often selected in patients with higher disease burden or specific clinical circumstances requiring rapid androgen suppression. Therefore, the observed elevated hazard ratio likely reflects underlying baseline disease characteristics and potential selection bias rather than a direct adverse oncologic effect of surgical castration. Moreover, the limited number of patients undergoing orchiectomy and the wide confidence interval necessitate careful interpretation. Current guidelines report comparable oncologic outcomes between surgical and medical castration [24].

We acknowledge that the high-risk subgroup comprised only 12 patients and the total number of events was limited (n = 33). In the conventional enter model, only PSA nadir retained significance, while SUVmax–IPI emerged as an independent predictor in the forward stepwise model. This discrepancy likely reflects overfitting in the enter model due to the large number of variables relative to events; the stepwise approach was chosen to identify the most robust predictors while preserving statistical stability. Nevertheless, the small subgroup size and the modest event number warrant cautious interpretation, and external validation in larger cohorts is essential.

A further limitation is that the inflammatory component of SUVmax–IPI may be influenced by non-malignant comorbidities such as coronary artery disease, hypertension, and diabetes mellitus. Although the prevalence of these conditions was balanced between SUVmax–IPI subgroups (Table 1), we were unable to quantify the inflammatory burden specifically attributable to each comorbidity. Therefore, we cannot completely exclude the possibility that the observed association partly reflects background systemic inflammation rather than tumor-related inflammation. Future studies should incorporate detailed assessments of metabolic syndrome, atherosclerotic burden, and other chronic inflammatory conditions to better disentangle these effects.

PSMA expression is known to be modulated by androgen-axis therapies. In our study, PSMA PET/CT was performed at diagnosis or before systemic treatment initiation in the vast majority of patients, which minimizes the influence of prior androgen-directed therapies. However, we did not systematically stratify patients by hormone-sensitive versus castration-resistant status, nor did we record the exact timing of androgen deprivation therapy initiation relative to imaging in every case. Moreover, subclinical androgen-axis modulation might still affect PSMA expression. These factors should be considered when interpreting single-time-point PSMA PET/CT parameters.

This study has several limitations. Its retrospective, single-center design and the relatively limited number of events may restrict generalizability. The cut-off value for SUVmax–IPI was determined using the Youden index within this cohort, raising the possibility of overfitting. Additionally, patients without available PSMA PET/CT imaging were excluded from SUVmax–IPI analysis, which may introduce selection bias, although baseline characteristics were broadly comparable.

Another limitation is that the IPI was calculated based on single-time-point measurements of CRP, albumin, and neutrophil-to-lymphocyte ratio. Given the dynamic nature of inflammatory markers, longitudinal assessment may further enhance prognostic performance. Prospective, multicenter validation studies are therefore warranted.

In conclusion, the SUVmax–IPI composite score integrating PSMA PET/CT-derived metabolic activity with systemic inflammatory burden appears to be a strong and independent prognostic biomarker for overall survival in metastatic prostate cancer. Our findings support the concept that integrated models combining tumor biology and host response may provide incremental prognostic information beyond conventional clinical and biochemical variables.

## 5. Conclusions

This study demonstrates that the SUVmax–IPI composite score, integrating PSMA PET/CT-derived tumor metabolic activity with systemic inflammatory burden, is independently associated with overall survival in metastatic prostate cancer. In the forward stepwise multivariable Cox model, SUVmax–IPI > 82 was associated with a nearly eight-fold increased risk of mortality (HR: 7.92; 95% CI: 2.97–21.10; *p* < 0.001), and patients with SUVmax–IPI ≤ 82 achieved a median overall survival of 125 months compared with only 19 months in those with SUVmax–IPI > 82 (log-rank *p* = 0.001). Compared with isolated PSMA PET/CT parameters and inflammatory indices, SUVmax–IPI provided more distinct prognostic stratification and retained its independence after adjustment for established clinical and biochemical markers, including ALP, AST, and PSA nadir. These findings suggest that combining metabolic tumor activity with host inflammatory response may enhance risk classification in metastatic prostate cancer. However, the cut-off value was determined within this cohort using the Youden index, which may lead to overfitting, and the retrospective, single-center design limits generalizability. The results should be interpreted in light of the small high-risk subgroup, the limited number of events, the potential of being confounding by comorbidities, and the dynamic nature of PSMA expression. Therefore, prospective multicenter validation is required before clinical implementation.

## Figures and Tables

**Table 1:** Baseline Demographic, Clinical, and Treatment Characteristics According to SUVmax–IPI Subgroups.

Variable		SUVmax–IPI ≤ 82 (n = 113) n (%)	SUVmax–IPI > 82 (n = 12) n (%)	*p* Value
Age at diagnosis, median (IQR)		68 (62.50–72.50)	65 (61.25–78.0)	0.775
Coronary artery disease		30 (26.5)	3 (25.0)	1.000
Hypertension		56 (49.6)	5 (41.7)	0.603
Diabetes mellitus		26 (23.0)	1 (9.1)	0.453
Smoking history		91 (80.5)	8 (66.6)	0.507
Benign prostatic hyperplasia		40 (35.4)	7 (58.3)	0.268
Family history of cancer		36 (31.9)	1 (8.3)	0.227
Gleason score at initial diagnosis	3 + 4/4 + 3	31 (27.5)	2 (18.2)	0.848
	4 + 4/3 + 5/5 + 3	15 (13.3)	1 (9.1)	
	4 + 5/5 + 4/5 + 5	66 (58.4)	8 (72.7)	
Clinical T stage at diagnosis	T1–T2	18 (16.0)	0 (0.0)	0.544
	T3	24 (21.2)	2 (16.7)	
	T4	67 (59.3)	9 (75.0)	
Regional lymph node positivity at diagnosis		82 (72.6)	8 (66.7)	0.230
Metastatic stage at diagnosis	M1a (non-regional lymph nodes only)	22 (21.0)	0 (0.0)	0.084
	M1b (bone metastasis)	56 (49.6)	10 (83.3)	
	M1c (visceral metastasis)	5 (4.8)	0 (0.0)	
Any local treatment to the primary tumor		38 (33.6)	3 (25.0)	0.949
Prior radical prostatectomy		25 (22.3)	2 (16.7)	0.627
Bone metastasis		86 (76.1)	12 (100.0)	0.068
Bone metastatic burden	1–3 lesions	17 (15.0)	2 (16.7)	0.154
	≥4 lesions (including ≥1 extra-axial)	69 (61.1)	10 (83.3)	
CHAARTED tumor volume	Low volume	38 (33.6)	2 (16.7)	0.231
	High volume	75 (66.4)	10 (83.3)	
Liver metastasis		9 (8.0)	0 (0.0)	0.598
Lung metastasis		10 (8.8)	1 (8.3)	1.000
Non-regional lymph node metastasis		96 (85.0)	10 (83.3)	0.925
First-line systemic therapy for metastatic disease	LHRH + abiraterone	31 (27.4)	2 (16.7)	0.743
	LHRH + enzalutamide	24 (21.2)	3 (25.0)	
	LHRH + docetaxel	35 (31.0)	6 (50.0)	
	LHRH + apalutamide	2 (1.8)	0 (0.0)	
	LHRH alone	17 (15.0)	1 (8.3)	
	LHRH + bicalutamide	4 (3.5)	0 (0.0)	
Response to first-line systemic therapy	Complete response	22 (19.5)	0 (0.0)	0.259
	Partial response	43 (38.1)	4 (33.3)	
	Stable disease	44 (38.9)	7 (58.3)	
	Progressive disease	4 (3.5)	1 (8.3)	
LHRH agent or orchiectomy	Goserelin	60 (53.1)	4 (33.3)	0.302
	Leuprolide acetate	50 (44.2)	7 (58.3)	
	Orchiectomy	3 (2.7)	1 (8.3)	
ECOG performance status at initiation of first-line therapy	ECOG 0–1	95 (84.1)	8 (66.7)	0.432
	ECOG ≥ 2	18 (15.9)	4 (33.3)	

Data are presented as n (%) or median (IQR). *p*-values were calculated using the chi-square or Fisher’s exact test for categorical variables and the Mann–Whitney U test for continuous variables. **Abbreviations:** SUVmax, maximum standardized uptake value; IPI, inflammatory prognostic index; LHRH, luteinizing hormone–releasing hormone; ECOG, Eastern Cooperative Oncology Group; CHAARTED, Chemohormonal Therapy Trial in Extensive Prostate Cancer.

**Table 2:** Univariate and multivariable Cox proportional hazards regression analyses for overall survival.

Variable	Univariate		Multivariate	
	HR (95% CI)	*p* Value	HR (95% CI)	*p* Value
ALP	1.002 (1.001–1.003)	0.001	1.002 (1.000–1.004)	0.032
AST	1.018 (0.999–1.038)	0.063	1.044 (0.997–1.094)	0.067
Albumin	0.459 (0.221–0.955)	0.037	1.229 (0.144–10.488)	0.850
LDH	1.004 (1.002–1.006)	<0.001	1.002 (0.998–1.006)	0.316
Hemoglobin	0.740 (0.624–0.878)	0.001	1.093 (0.770–1.550)	0.619
Liver metastasis	3.573 (1.216–10.503)	0.021		
Lung metastasis	0.368 (0.140–0.972)	0.044		
ECOG performance status ≥ 2 (vs. 0–1)	2.86 (1.39–5.87)	0.004		
PSA nadir level	1.006 (1.004–1.009)	<0.001		
SUVmax–IPI	1.003 (1.002–1.005)	<0.001		

Hazard ratios (HRs) with 95% confidence intervals (CIs) and corresponding *p*-values are presented. The full univariate and multivariable analyses are provided in Supplementary Table S1. Abbreviations: HR, hazard ratio; CI, confidence interval; ALP, alkaline phosphatase; AST, aspartate aminotransferase; LDH, lactate dehydrogenase; PSA, prostate-specific antigen; ECOG, Eastern Cooperative Oncology Group; IPI, inflammatory prognostic index.

**Table 3:** Final Forward Stepwise Multivariable Cox Proportional Hazards Model for Overall Survival.

Variable	HR 95% CI	*p* Value
ALP	1.002 (1.000–1.004)	0.032
AST	1.031 (1.002–1.060)	0.035
PSA nadir	1.006 (1.004–1.009)	<0.001
LHRH therapy (ref: goserelin)		0.011
Leuprolide acetate	0.85 (0.37–1.95)	0.703
Orchiectomy	10.18 (2.06–50.17)	0.004
SUVmax–IPI > 82	7.917 (2.971–21.098)	<0.001

Hazard ratios (HRs) with 95% confidence intervals (CIs) and corresponding *p*-values are presented. Abbreviations: HR, hazard ratio; CI, confidence interval; ALP, alkaline phosphatase; AST, aspartate aminotransferase; PSA, prostate-specific antigen; LHRH, luteinizing hormone–releasing hormone; IPI, inflammatory prognostic index.

**Table 4:** Laboratory and PSMA PET/CT Findings According to SUVmax–IPI Subgroups.

Variable	SUVmax–IPI ≤ 82 (n = 113) Median (IQR)	SUVmax–IPI > 82 (n = 12) Median (IQR)	*p*-Value
ALP (U/L)	90 (69.5–134.0)	182.5 (122.5–504.5)	0.001
CRP (mg/L)	0.30 (0.14–0.80)	6.75 (5.43–9.63)	<0.001
Albumin (g/dL)	4.10 (3.90–4.30)	3.75 (3.53–3.90)	0.001
LDH (U/L)	208 (178–250)	263.5 (204.5–400.3)	0.014
Absolute neutrophil count (×10^3^/µL)	4.600 (3.635–5.920)	5.855 (4.690–6.815)	0.031
Hemoglobin (g/dL)	13.3 (12.0–14.4)	11.0 (8.53–12.23)	<0.001
PSA at metastasis (ng/mL)	46.0 (7.95–134.5)	127.0 (33.3–472.0)	0.028
PSA nadir (ng/mL)	0.30 (0.01–2.05)	1.14 (0.23–6.20)	0.035
Metastatic lesion SUVmax	14.70 (5.80–28.05)	28.80 (16.15–45.50)	0.012
Metastatic total lesion PSMA (TL-PSMA)	29.30 (11.40–92.25)	206.85 (45.88–279.98)	<0.001

Continuous variables are presented as median (interquartile range, IQR). *p*-values were calculated using the Mann–Whitney U test. Abbreviations: ALP, alkaline phosphatase; CRP, C-reactive protein; LDH, lactate dehydrogenase; PSA, prostate-specific antigen; PSMA, prostate-specific membrane antigen; TL-PSMA, total lesion PSMA; IQR, interquartile range.

## Data Availability

The datasets generated and/or analyzed during the current study are available from the corresponding author on reasonable request.

## Supplementary Materials

The following supporting information can be downloaded at: https://www.mdpi.com/article/10.3390/jcm15072655/s1, Table S1: Univariate and multivariable Cox regression analyses for overall survival (full model); Table S2: Laboratory and PSMA PET/CT parameters according to SUVmax–IPI subgroups (complete analysis).

## Abbreviations

The following abbreviations are used in this manuscript:

**Abbreviation**	**Definition**
ADT	Androgen Deprivation Therapy
ALB	Albumin
ALB/ALP	Albumin-to-Alkaline Phosphatase Ratio
ALP	Alkaline Phosphatase
AST	Aspartate Aminotransferase
AUC	Area Under the Curve
CHAARTED	ChemoHormonal Therapy versus Androgen Ablation Randomized Trial for Extensive Disease in Prostate Cancer
CI	Confidence Interval
CRP	C-Reactive Protein
CT	Computed Tomography
ECOG	Eastern Cooperative Oncology Group
HR	Hazard Ratio
IBI	Immune-Based Prognostic Index
IPI	Inflammatory Prognostic Index
IQR	Interquartile Range
LDH	Lactate Dehydrogenase
LHRH	Luteinizing Hormone–Releasing Hormone
NLR	Neutrophil-to-Lymphocyte Ratio
OS	Overall Survival
PET/CT	Positron Emission Tomography/Computed Tomography
PSA	Prostate-Specific Antigen
PSMA	Prostate-Specific Membrane Antigen
PSMA-TV	PSMA Tumor Volume
ROC	Receiver Operating Characteristic
SE	Standard Error
SIRI	Systemic Inflammation Response Index
SUVmax	Maximum Standardized Uptake Value
TL-PSMA	Total Lesion PSMA
TNM	Tumor–Node–Metastasis
