# Supplementary material for: Prognostic Value of the SUVmax–IPI Composite Score on Overall Survival in Metastatic Prostate Cancer
Source: J Clin Med. 2026 Mar 31;15(7):2655. doi: 10.3390/jcm15072655 (PMC13072720; doi:10.3390/jcm15072655)
Supplement: Supplementary file 1 [file jcm-15-02655-s001.zip › jcm-4198030-supplementary.pdf]

Table S1. Full univariable and multivariable Cox proportional hazards regression analyses for overall survival.

Variable		Univariate		Multivariate	
		HR (95% CI)	P value	HR (95% CI)	P value
Age at diagnosis		1.012 (0.966-1.059)	0.621		
Coronary artery disease		1.715 (0.840-3.503)	0.139		
Hypertension		1.383 (0.686-2.789)	0.364		
Diabetes mellitus		0.835 (0.352-1.982)	0.683		
Smoking status (ref: never smoker)	Current smoker	0.965 (0.421-2.210)	0.932		
	Former smoker	0.576 (0.219-1.519)	0.265		
ALP		1.002 (1.001-1.003)	0.001	1.002 (1.000-1.004)	0.032
AST		1.018 (0.999-1.038)	0.063	1.044 (0.997-1.094)	0.067
ALT		1.004 (0.976-1.032)	0.791		
Creatinine		1.246 (0.777-1.999)	0.361		
Estimated glomerular filtration rate (eGFR)		0.993 (0.979-1.007)	0.339		
CRP		1.065 (0.986-1.151)	0.111		
Total serum bilirubin		0.537 (0.127-2.279)	0.399		
Albumin		0.459 (0.221-0.955)	0.037	1.229 (0.144-10.488)	0.850
LDH		1.004 (1.002-1.006)	<0.001	1.002 (0.998-1.006)	0.316
Absolute neutrophil count		1.000 (1.000-1.000)	0.321		
Hemoglobin		0.740 (0.624-0.878)	0.001	1.093 (0.770-1.550)	0.619
Platelet count		1.000 (1.000-1.000)	0.233		
PSA at diagnosis		1.001 (0.999-1.003)	0.228		
Gleason score at diagnosis (ref: 3+4)			0.929		
	4+3	0.588 (0.147-2.355)	0.453		
	4+4, 3+5, 5+3	0.568 (0.138-2.334)	0.433		
	4+5, 5+4, 5+5	0.560 (0.162-1.934)	0.359		
Liver metastasis		3.573 (1.216-10.503)	0.021		
Lung metastasis		0.368 (0.140-0.972)	0.044		
T stage at diagnosis (ref: T1)	T2	1.915 (0.220-16.672)	0.556		
	T3	1.657 (0.194-14.141)	0.645		
	T4	1.716 (0.225-13.086)	0.602		
N stage at diagnosis (ref:N0)	Regional lymph node(s)	0.925 (0.337-2.536)	0.703		
Metastatic stage at diagnosis(Ref: No metastasis)			0.679		
	M1a (non-regional LN only)	0.689 (0.207-2.292)	0.543		
	M1b (bone metastasis)	0.643 (0.264-1.563)	0.330		
	M1c (visceral metastasis)	1.455 (0.350-6.059)	0.606		
ECOG performance status ≥2 (vs. 0–1)		2.86 (1.39-5.87)	0.004		
PSA nadir level		1.006 (1.004-1.009)	<0.001		
Prostate SUVmax		0.998 (0.976-1.021)	0.886		
Prostate PSMA tumor volume (PSMA-TV)		0.992 (0.961-1.023)	0.604		
Prostate total lesion PSMA (TL-PSMA)		1.000 (0.998-1.001)	0.758		
Metastatic SUVmax		1.007 (0.996-1.018)	0.203		
Metastatic PSMA tumor volume (PSMA-TV)		1.000 (0.963-1.038)	0.985		
Metastatic total lesion PSMA (TL-PSMA)		1.001 (0.999-1.002)	0.511		
Systemic inflammation response index (SIRI)		1.000 (1.000-1.000)	0.765		
Inflammatory burden index (IBI)		1.000 (0.995-1.006)	0.906		
De Ritis ratio (AST/ALT ratio)		1.348 (0.951-1.909)	0.093		
Inflammatory prognostic index (IPI)		0.999 (0.983-1.015)	0.917		
SUVmax–IPI		1.003 (1.002-1.005)	<0.001		

Hazard ratios (HRs) with 95% confidence intervals (CIs) and corresponding p-values are presented. All candidate variables evaluated in the full model are included. Abbreviations: HR, hazard ratio; CI, confidence interval.

Table S2. Laboratory and PSMA PET/CT parameters according to SUVmax–IPI subgroups (full analysis).

Variable	SUVmax–IPI ≤82 (n=113), Median (IQR)	SUVmax–IPI >82 (n=12), Median (IQR)	p-value
ALP (U/L)	90 (69.5–134.0)	182.5 (122.5–504.5)	0.001
AST (U/L)	21 (17–26)	21 (15.3–30.5)	0.916
ALT (U/L)	16 (12.0–22.0)	15.5 (12.3–19.3)	0.600
Creatinine (mg/dL)	0.83 (0.70–1.04)	0.97 (0.63–1.39)	0.513
eGFR (mL/min/1.73 m^2)	88 (71.5–98.0)	81 (48.3–103.0)	0.492
CRP (mg/L)	0.30 (0.14–0.80)	6.75 (5.43–9.63)	<0.001
Total serum bilirubin (mg/dL)	0.50 (0.40–0.78)	0.59 (0.40–0.94)	0.428
Albumin (g/dL)	4.10 (3.90–4.30)	3.75 (3.53–3.90)	0.001
LDH (U/L)	208 (178–250)	263.5 (204.5–400.3)	0.014
Absolute neutrophil count (×10^3/μL)	4.600 (3.635–5.920)	5.855 (4.690–6.815)	0.031
Absolute lymphocyte count (×10^3/μL)	1.880 (1.380–2.315)	1.690 (1.293–1.945)	0.151
Hemoglobin (g/dL)	13.3 (12.0–14.4)	11.0 (8.53–12.23)	<0.001
Platelet count (×10^3/μL)	233 (200.5–281.5)	256.5 (137.0–329.5)	0.763
PSA at diagnosis (ng/mL)	53 (12.6–122.5)	115 (32.8–277.5)	0.058
PSA at metastasis (ng/mL)	46.0 (7.95–134.5)	127.0 (33.3–472.0)	0.028
PSA nadir (ng/mL)	0.30 (0.01–2.05)	1.14 (0.23–6.20)	0.035
Prostate SUVmax	12.85 (5.93–23.55)	7.95 (4.78–18.10)	0.253
Prostate PSMA tumor volume (PSMA-TV)	11.10 (4.65–19.15)	11.80 (6.85–24.40)	0.394
Prostate total lesion PSMA (TL-PSMA)	61.50 (23.80–157.00)	71.70 (21.58–337.18)	0.484
Metastatic lesion SUVmax	14.70 (5.80–28.05)	28.80 (16.15–45.50)	0.012
Metastatic PSMA tumor volume (PSMA-TV)	5.10 (2.55–12.45)	9.10 (4.30–19.33)	0.122
Metastatic total lesion PSMA (TL-PSMA)	29.30 (11.40–92.25)	206.85 (45.88–279.98)	<0.001

Continuous variables are presented as median (interquartile range, IQR). P-values were calculated using the Mann–Whitney U test. Abbreviations: ALP, alkaline phosphatase; AST, aspartate aminotransferase; ALT, alanine aminotransferase; eGFR, estimated glomerular filtration rate; CRP, C-reactive protein; LDH, lactate dehydrogenase; PSA, prostate-specific antigen; PSMA, prostate-specific membrane antigen; TV, tumor volume; TL-PSMA, total lesion PSMA; IQR, interquartile range.
